# Estimating the cost of illness and burden of disease associated with the 2014–2015 chikungunya outbreak in the U.S. Virgin Islands

**DOI:** 10.1371/journal.pntd.0007563

**Published:** 2019-07-19

**Authors:** Leora R. Feldstein, Esther M. Ellis, Ali Rowhani-Rahbar, Morgan J. Hennessey, J. Erin Staples, M. Elizabeth Halloran, Marcia R. Weaver

**Affiliations:** 1 Department of Epidemiology, University of Washington School of Public Health, Seattle, Washington, United States of America; 2 Vaccine and Infectious Disease Division, Fred Hutchinson Cancer Research Center, Seattle, Washington, United States of America; 3 U.S. Virgin Islands Department of Health, Saint Croix, United States Virgin Islands, United States of America; 4 Division of Vector-Borne Diseases, National Center for Emerging and Zoonotic Infectious Diseases, Centers for Disease Control and Prevention, Fort Collins, Colorado, United States of America; 5 Center for Inference and Dynamics of Infectious Diseases, Fred Hutchinson Cancer Research Center, Seattle, Washington, United States of America; 6 Department of Biostatistics, University of Washington School of Public Health, Seattle, Washington, United States of America; 7 Departments of Health Metrics Sciences and Global Health, Institute for Health Metrics and Evaluation, University of Washington, Seattle, Washington, United States of America; McGill University, CANADA

## Abstract

Chikungunya virus (CHIKV), an alphavirus that causes fever and severe polyarthralgia, swept through the Americas in 2014 with almost 2 million suspected or confirmed cases reported by April 2016. In this study, we estimate the direct medical costs, cost of lost wages due to absenteeism, and years lived with disability (YLD) associated with the 2014–2015 CHIKV outbreak in the U.S. Virgin Islands (USVI). For this analysis, we used surveillance data from the USVI Department of Health, medical cost data from three public hospitals in USVI, and data from two studies of laboratory-positive cases up to 12 months post illness. On average, employed case-patients missed 9 days of work in the 12 months following their disease onset, which resulted in an estimated cost of $15.5 million. Estimated direct healthcare costs were $2.9 million for the first 2 months and $0.6 million for 3–12 months following the outbreak. The total estimated cost associated with the outbreak ranged from $14.8 to $33.4 million (approximately 1% of gross domestic product), depending on the proportion of the population infected with symptomatic disease, degree of underreporting, and proportion of cases who were employed. The estimated YLDs associated with long-term sequelae from the CHIKV outbreak in the USVI ranged from 599–1,322. These findings highlight the significant economic burden of the recent CHIKV outbreak in the USVI and will aid policy-makers in making informed decisions about prevention and control measures for inevitable, future CHIKV outbreaks.

## Author summary

Chikungunya, a virus carried and transmitted by mosquitoes, causes fever, headache, and severe joint pain in humans that often resolves within 7–10 days. However, a proportion of cases, up to 79% in some outbreaks, report persistent joint pain and chronic inflammatory rheumatism, resulting in decreased quality of life for months to years following initial infection. In 2014, chikungunya virus swept through the Americas, resulting in almost 2 million suspected or confirmed cases reported by April 2016. Previous studies have noted the large resource burden from chikungunya outbreaks, including high healthcare costs, lost wages due to absenteeism, and decreased quality of life for months following infection. Our work aimed to estimate the direct medical costs, cost of lost productivity due to absenteeism, and years lived with disability associated with the chikungunya outbreak in the U.S. Virgin Islands. This information may aid policy-makers in making informed decisions about prevention and control measures for inevitable, future chikungunya outbreaks.

## Introduction

Chikungunya virus (CHIKV), an alphavirus transmitted by *Aedes (Stegomyia)* species mosquitoes, was introduced into the Americas in December of 2013 [1]. By April 2016, almost 2 million suspected or confirmed cases were reported in 45 countries and territories in the Caribbean, Central, South, and North America [2,3]. Acute symptoms, including high fever, severe polyarthralgia, headache and myalgia, often resolve within 7–10 days [4–6]. However, a proportion of cases, up to 79% in some outbreaks, report persistent arthralgia and chronic inflammatory rheumatism, resulting in decreased quality of life for months to years following initial infection [5–16]. Currently, there is no antiviral treatment or vaccine for the infection, there are no specific therapeutics for chronic symptoms, and public health prevention measures, such as mosquito reduction, have thus far proven to be insufficient [4,17].

CHIKV was first identified to be locally transmitted in the U.S. Virgin Islands (USVI) in June 2014. By February 2015, almost 2,000 suspected cases had been reported in a population of 103,574 people [18,19]. The epidemiology of the CHIKV outbreak in the USVI has been previously described [20]. Previous studies from CHIKV outbreaks in La Réunion, Colombia, and India have noted the large resource burden from these outbreaks including high healthcare costs, lost wages due to absenteeism, and decreased quality of life for months following infection [21–26]. To our knowledge, the economic impact of the recent CHIKV epidemic in the Caribbean and years lived with disability (YLDs) associated with long-term sequelae of CHIKV illness have not been quantified. This information would inform decisions about prevention and control measures for inevitable, future CHIKV outbreaks. Using a societal perspective, we aim to estimate the cost of illness and burden of disease associated with the 2014–2015 CHIKV outbreak in the USVI by estimating direct medical costs, indirect cost of lost productivity due to absenteeism, and YLDs associated with long-term sequelae of the outbreak.

## Methods

### Ethics statement

Verbal informed consent was obtained from all participants before interviewing them. Parental/guardian consent was acquired on behalf of all child participants and parents/guardians responded for children under the age of 12. Verbal informed consent was documented on the questionnaire by the interviewer and entered into the database. Oral consent was used because almost half of the interviews took place over the phone. Ethics approval for this study, as well as the use of verbal consent was obtained from the University of the Virgin Islands and the University of Washington.

### Study populations and data inputs

Estimates of the direct and indirect cost of the outbreak were based on suspected cases reported to USVI Department of Health (DOH). All costs were expressed in 2014 U.S. dollars (USD). A suspected case was defined as a resident of the USVI who visited a hospital or healthcare clinic on St. John, St. Thomas, or St. Croix with acute onset of fever (≥38°C) and severe arthralgia or arthritis not explained by another medical condition. A laboratory-positive case was defined as a suspected case whose blood sample tested positive for either CHIKV RNA or IgM antibodies. Of all reported suspected CHIKV cases who were tested, 30% tested negative for CHIKV. Therefore, when we used surveillance data to estimate potential costs, we used 0.70 as the proportion of non-tested reported suspected CHIKV cases who would have been positive had they been tested.

Laboratory-positive cases were contacted by telephone and invited to participate in a follow-up investigation at 1–2, 6 and 12 months after the acute phase of illness, as previously defined (S1 Table) [27]. The 1 to 2-month questionnaire asked about hospitalization and healthcare utilization during the first months after initial infection. The 12-month questionnaire asked additional questions about use of prescription medication and healthcare utilization between the first and last interview.

Estimates of YLDs were based on reports of persistent arthralgia. Similar to a previous study [21], we defined persistent arthralgia as joint pain at least once per week that occurred more than 15 days after the acute phase of illness. We used data from two previous studies to determine YLDs. The first study assessed the proportion of persons with laboratory-positive CHIKV infection who reported persistent arthralgia compared to a non-symptomatic control group of individuals who visited an emergency room of a hospital or a health care clinic in the USVI and were interviewed regarding presence of persistent arthralgia [27]. The control group was defined as USVI residents who did not report experiencing sudden onset of fever and joint pain in June 2014-June 2015. The second study was a population-based study of seroprevalence that assessed the frequency of persistent arthralgia approximately 12 months following the introduction of the CHIKV and determined the proportion of persistent arthralgia attributable to CHIKV infection [28].

### Estimating indirect costs

Productivity lost per CHIKV case was estimated assuming a standard 40-hour work week, and using the average hourly wage for each island [29]. Average hourly wages from the USVI were not available by gender or age. The following formula was used to estimate value of time lost due to CHIKV disease:

Valueoftimelost=Mean#ofworkdaysmissedateachtimepoint*8hoursperday*averagehourlywage*(total#ofreportedlaboratory-positiveCHIKVcases+0.70*#ofnon-testedreportedsuspectedCHIKVcases)

where mean # of work days missed include both market and non-market productivity. To obtain an estimate of the total wages lost for cases who were not reported, we used data from a 2015 seroprevalence study in the USVI that found an infection rate of 31%, (95% CI: 26%–36%), with 72% of those infected reporting symptomatic infection [28]. Based on this information, we estimated the fraction of the population with symptomatic infection to be 22% (0.31 * 0.72). The estimated number of symptomatic CHIKV infections in the USVI population was multiplied by productivity lost per person to obtain an overall cost estimate of absenteeism due to the outbreak. This estimate assumes that absenteeism from school and other non-market activities has the same monetary value as formal employment. In reviewing both CHIKV and dengue cost-of-illness methodologies, some studies included all individuals with the disease or condition regardless of employment status (to capture overall loss of productivity), while others included only those who were officially employed [22,23,25,30–38]. As a sensitivity analysis, we calculated absenteeism associated with CHIKV illness for only those who were employed (52.2% of the USVI population as of 2010) [39]. Because the 2015 serosurvey estimated that 70% of symptoms (acute fever and joint pain) among CHIKV infected individuals were attributable to their infection, we also conducted a sensitivity analysis to estimate the cost of absenteeism when including only the proportion of individuals with symptoms directly attributable to CHIKV infection (0.31 * 0.72 * 0.70 = 0.16) [26].

### Estimating direct medical costs

The medical costs for two phases of the illness (acute and long-term) were estimated with two different sources of data. For the acute phase of illness, inpatient and outpatient charges of all suspected CHIKV cases from Governor Juan F. Luis Hospital and Medical Center (JFLHMC), the public hospital in St. Croix, were obtained from the finance department of the hospital. Mean costs of inpatient and outpatient visits among reported cases were calculated separately and multiplied by the total number of inpatient and outpatient visits captured by the USVI DOH surveillance system. Calculation assumes standard of care was the same across hospitals. These costs were applied to patients on all three islands, because cost data for suspected CHIKV cases were unavailable from Schneider Regional Medical Center (SRMC) in St. Thomas and Myra Keating Community Health Center (MKCHC) in St. John, the other two public healthcare facilities in the USVI. A sensitivity analysis was conducted for the missing cost data from SRMC and MKCHC based on the mean cost of standard outpatient and inpatient visits from those two healthcare facilities (S2 Table). Data on diagnosis codes and length of inpatient stay were not collected.

For the cost of subsequent outpatient visits up to 12 months after illness onset, the mean cost of standard outpatient visit was obtained from the finance departments of JFLHMC, SRMC and MKCHC. The mean number of additional healthcare visits reported by cases for treatment of CHIKV after acute illness from the interview sample was calculated from the 1–2 and 12-month questionnaires. The mean number of visits was multiplied by the total number of reported laboratory-positive cases and 70% of suspected but not tested cases by island to obtain an overall estimate of additional healthcare costs up to 12 months after acute illness. Note that these calculations are limited to reported cases, assuming that only people who sought healthcare at 1–2 months after the outbreak would seek follow-up care.

Current literature indicates that a recall period of 1–2 months provides reliable estimates for outpatient visits [40–43]; however, previous studies have shown that 5%–47% of visits were not reported when individuals were interviewed about healthcare utilization of physician visits during a 12 month recall period [44,45], while other studies have shown no underreporting [46]. Due to potential underreporting of healthcare utilization 12 months after illness onset, a sensitivity analysis was performed using a range of underreporting from 5–47% (S3 Table).

### Estimating YLDs

Prior studies estimating YLDs for CHIKV have used disability weights for osteoarthritis and rheumatoid arthritis since a disability weight has not been assigned to CHIKV disease [22,24,26,47]. However, these weights are from the 1990 Global Burden of Disease [48]. Here, we use the disability weight for post-acute effects from infectious diseases from the 2013 Global Burden of Disease study [49], and use the weights for osteoarthritis and rheumatoid arthritis as a sensitivity analysis to maintain consistency with previous studies.

We calculated YLDs to estimate the amount of time, ability, and activity lost due to persistent arthralgia from CHIKV illness using the following equation [50]:

YLD=(Disabilityweight*NumberofsymptomaticCHIKVinfectionsintheUSVI*Prevalenceofpersistentarthralgia6monthsafteracuteillnessonset*182.625/365.25)+(Disabilityweight*NumberofsymptomaticCHIKVinfectionsintheUSVI*Prevalenceofpersistentarthralgia12monthsafteracuteillnessonset*182.625/365.25)

The number of symptomatic CHIKV infections in the USVI is based on an estimate from the 2015 serosurvey in the USVI [28]. To ensure that reported persistent arthralgia among cases was due to CHIKV and not from other causes, we used a 32% prevalence estimate of persistent arthralgia among CHIKV cases interviewed at 6 months and a 21% prevalence estimate of persistent arthralgia among CHIKV cases interviewed at 12 months: 44% at 6 months and 33% at 12 months net of the 12% prevalence of persistent arthralgia in the non-symptomatic control group [27]. This latter estimate is consistent with the prevalence of reported arthritis in the USVI population from the Behavioral Risk Factor Surveillance System Report (15%) [39]. We also used a more conservative 12-month estimate of persistent arthralgia attributable to CHIKV from the 2015 serosurvey in the USVI of 12% (95% CI: 7–17%) [28]. The serosurvey did not assess persistent arthralgia at 6 months. Years of life lost were not calculated because cause of death could not be determined for the three deceased suspected CHIKV cases.

## Results

### Impact of CHIKV outbreak in USVI

One to two months after acute disease onset, 86 laboratory-positive CHIKV cases were interviewed. Of the cases who were employed (33%), 89% reported missing work due to CHIKV illness (Table 1). On average, employed cases reported missing 6 days of work within 1–2 months after onset of CHIKV symptoms. One to two months after their initial healthcare visit, 33% of cases reported seeking additional healthcare at a clinic after initial infection and 9% reported being hospitalized due to CHIKV illness.

**Table 1:** Percentage of laboratory-positive cases 1–2, 6, and 12 months after disease onset who missed work, daily activities/chores, sought additional healthcare, were hospitalized due to chikungunya (CHIKV) illness and prescribed medication for CHIKV, U.S. Virgin Islands.

Interview date	1–2 Month (n = 86)			3–6 Month (n = 165)			7–12 Month (n = 128)		
**Employment Status**	**% (n)**	**Median (range)**	**Mean**	**% (n)**	**Median (range)**	**Mean**	**% (n)**	**Median (range)**	**Mean**
Working	33 (28)	-	-	41 (67)	-	-	34 (43)	-	-
Child/Student	24 (21)	-	-	16 (26)	-	-	23 (30)	-	-
**Missed work/school**									
Working (days)	89 (25)	4.5 (0–21)	5.6	88 (58)	0.5 (0–60)	2.2	9 (4)	0 (0–40)	1.2
Child/Student (days)	53 (10)*	1.0 (0–7)	1.6	62 (16)	2.3 (0–20)	3.4	7 (2)	0 (0–60)	2.1
**Missed daily activities/chores** (days)	86 (61)	5 (0–62)	11.7	86 (135)	5.0 (0–140)	13.0	15 (19)	0 (0–168)	6.4
**Additional healthcare** (visits)	33 (28)	0 (0–6)	0.5	-	-	-	25 (34)	0 (0–17)	0.6
**Hospitalization**	9 (8)	0 (0–14)	0.4	-	-	-	-	-	-
**Prescribed medication**	-	-	-	-	-	-	24.19 (30)	-	-

*Many of the students interviewed at the 1 to 2-month follow-up were on summer vacation when they became ill with CHIKV and therefore the number of school days missed is lower than what might be expected if the outbreak occurred during the school year.

Six months after acute disease onset, 165 laboratory-positive CHIKV cases were interviewed. Of the cases who were employed (41%), 88% reported missing work due to CHIKV illness, 4–5 months after their 1–2 month interview (Table 1). On average, employed cases reported missing two additional days of work 4–5 months after the 1–2 month interview.

Twelve months after acute disease onset, 128 of the 165 laboratory-positive CHIKV cases were interviewed. Of the cases who were employed (34%), 9% reported missing work due to CHIKV illness during the six months after their 6-month interview (Table 1). On average, employed cases reported missing one additional day of work during that time period. Of the interviewed cases, 25% reported seeking additional healthcare 10–11 months after the 1–2 month interview and 24% reported taking prescription medication in the last 12 months for CHIKV-related symptoms. Forty percent (n = 12) of those who reported taking prescription medication indicated that they were prescribed prednisone for joint pain and 47% (n = 14) reported taking prescribed opioids for joint pain.

### Indirect cost estimate

**Fig 1:**
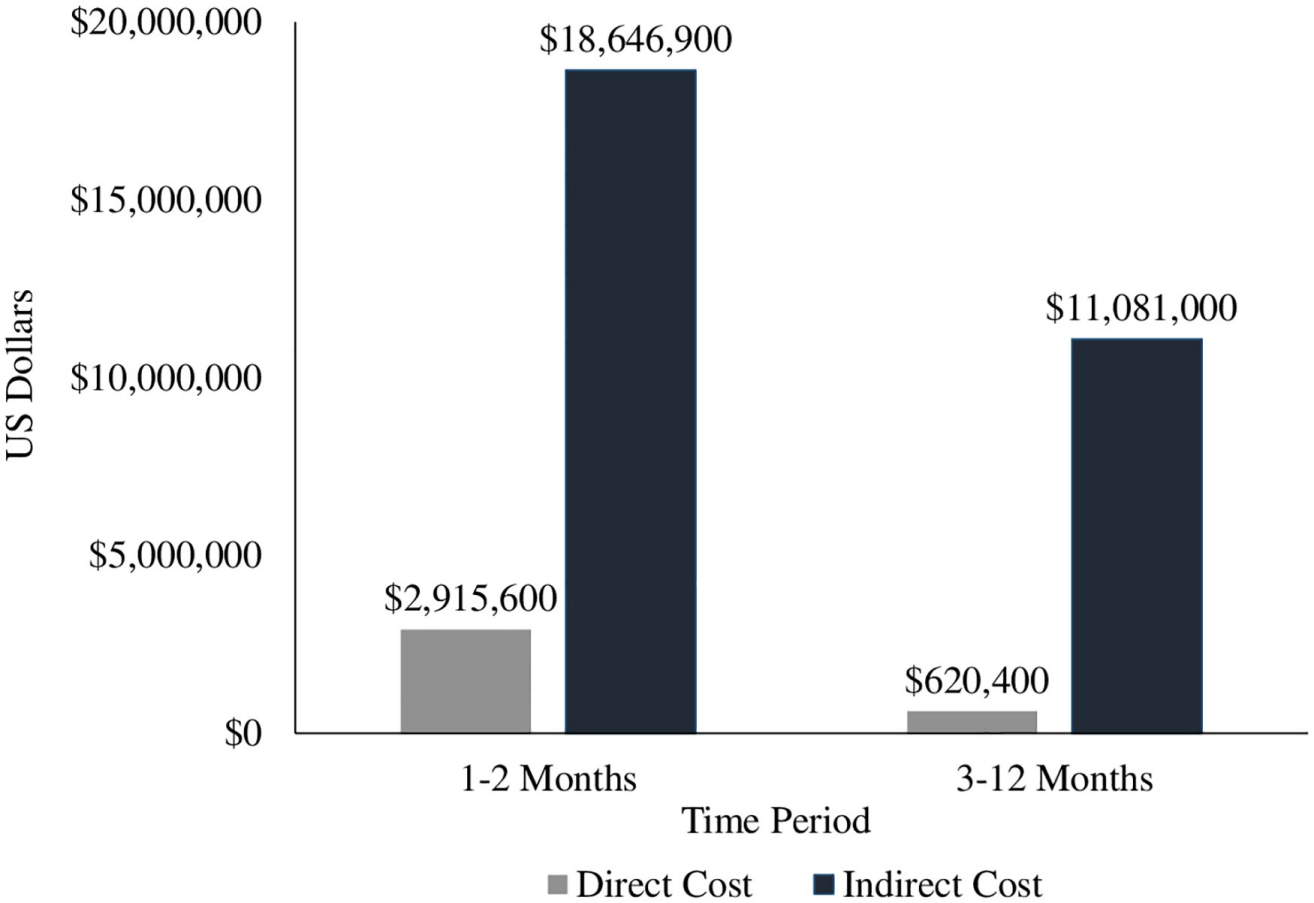
Total direct and indirect cost estimate (2014 USD) of the chikungunya outbreak in the U.S. Virgin Islands up to 12 months after illness onset.

The average cost of absenteeism related to CHIKV disease 1–2 months after illness onset ranged from $713–$825 per person, depending on island of residence (Table 2). Six months after illness onset, the average cost of absenteeism ranged from $275–$318 per person and 12 months after illness onset, the average cost per person ranged from $148-$172. The total estimated cost of absenteeism associated with acute and long-term CHIKV illness up to 12 months after CHIKV disease onset was $1.76 million for all reported laboratory-positive cases and 70% of all suspected but not tested CHIKV cases. However, when using the estimated proportion of symptomatic CHIKV infection in the USVI (0.22), almost 12 times the number of individuals were infected with CHIKV than were captured by surveillance data. When including these additional cases, the total estimated cost of absenteeism for acute and long-term CHIKV illness up to 12 months after CHIKV disease onset was $29.7 million (Table 2 & Fig 1). The total estimated cost of absenteeism associated with acute and long-term CHIKV illness up to 12 months after CHIKV disease onset for only the USVI population that was employed (52%) was $15.5 million but this figure does not account for absenteeism from school and other non-market activities. Among infected individuals with symptoms attributable to CHIKV (0.16), the estimated cost of absenteeism associated with acute and long-term CHIKV illness up to 12 months after CHIKV disease onset was $21.6 million, and $11.3 million when including only the proportion of the USVI population who was employed (S4 Table).

**Table 2:** Indirect cost estimates (2014 USD) due to absenteeism from the chikungunya outbreak in the U.S. Virgin Islands up to 12 months after disease onset.

Time period after acute illness	1–2 Months			3–6 Months			7–12 Months		
Median number of work days missed	4.5			0.5			0		
Mean number of work days missed	5.6			2.2			1.2		
Mean number of work hours missed	44.6			17.2			9.3		
**Island**	St. Thomas	St. Croix	St. John	St. Thomas	St. Croix	St. John	St. Thomas	St. Croix	St. John
Average Hourly Wage ($) [29]	18.51	18.43	16.00	18.51	18.43	16.00	18.51	18.43	16.00
Wages lost per case by island ($)	824.81	821.24	712.96	318.37	317.00	275.20	171.77	171.03	148.48
**Reported cases**									
Number of reported laboratory-positive cases + 70% of suspected not-tested cases	804	508	34	804	508	34	804	508	34
Total value of time lost by island for reported cases ($)	663,147	417,190	24,241	255,969	161,036	9,357	138,103	86,883	5,048
**Indirect cost of the CHIKV outbreak for reported cases**			**1,761,000**						
Total wages lost by island for employed*, reported cases ($)	346,163	217,773	12,654	133,616	84,061	4,884	72,090	45,353	2,635
**Indirect cost of the CHIKV outbreak for reported cases who are employed**			**919,200**						
**Estimated cases**									
Number of estimated cases by island when proportion of population with symptomatic infection = 0.22	11,051	10,835	889	11,051	10,835	889	11,051	10,835	889
Total value of time lost by island when proportion of population with symptomatic infection = 0.22 ($)	9,114,975	8,898,135	633,821	3,518,307	3,434,695	244,653	1,898,230	1,853,110	131,999
**Indirect cost of the CHIKV outbreak of for complete USVI population ($)**	**29,72,900**								
Total wages lost by island for among all employed when proportion of population with symptomatic infection = 0.22 ($)	4,758,017	4,644,827	330,855	1,836,556	1,792,911	127,709	990,876	967,323	68,903
Indirect cost of the CHIKV outbreak for USVI population reported to be employed* **($)**	**15,518,000**								

*52% of the U.S. Virgin Islands population was employed as of 2010 [39].

Note: Total cost estimates were rounded to the nearest hundred.

### Direct cost estimate: Acute phase of illness

The average cost of an outpatient visit for a suspected CHIKV case during the acute phase of illness was $1,526 and the average cost of an inpatient visit was $16,982 (Table 3). These costs include laboratory testing and prescription medication. Of the 1,929 reported suspected cases, 1,850 had outpatient visits and 79 suspected cases were hospitalized. Assuming that 70% of these suspected cases were laboratory-positive, the total estimated cost of outpatient and inpatient healthcare visits associated with suspected CHIKV cases during the acute phase of the outbreak was $2.9 million, with the hospitalized cases comprising 48% of the total cost. As shown from the sensitivity analysis in S2 Table, adjusting the direct costs by the relative average outpatient cost reduces the total estimated direct cost by 27%.

**Table 3:** Direct cost estimate (2014 USD) of the chikungunya outbreak in the U.S. Virgin Islands up to 2 months after illness onset, based on cost estimates from St. Croix.

Outpatient		Inpatient	
Median cost of an outpatient healthcare visit ($)	1,365	Median cost of an inpatient healthcare visit ($)	14,551
Mean cost of an outpatient healthcare visit ($)	1,526	Mean cost of an inpatient visit ($)	16,983
Total number of outpatient reported suspected cases * 70% of suspected not-tested cases	1,295	Total number of inpatient reported suspected cases * 70% of suspected not-tested cases	55
Total cost of outpatient visits related to CHIKV ($)	1,976,442	Total cost of inpatient visits related to CHIKV	939,145
**Total cost of outpatient and inpatient visits related to CHIKV ($)**	**2,915,600**		

Note: Total cost estimate was rounded to the nearest hundred.

### Direct cost estimate: Up to 12 months after acute phase of illness

The 86 CHIKV cases interviewed 1–2 months after acute illness reported, on average, having 0.5 additional healthcare visits related to CHIKV disease (Table 4). The average cost of a standard outpatient visit varied by healthcare facility and island but ranged from $234-$600. The 128 CHIKV cases interviewed 12 months after acute illness reported having on average 0.62 additional healthcare visits related to CHIKV disease 10–11 months after their 1–2 month interview. Therefore, the total estimated cost of additional outpatient healthcare visits related to CHIKV disease up to one year after illness onset was $620,400 (Table 4 & Fig 1). The sensitivity analysis for the potential underreporting of healthcare utilization 12 months after illness onset provided the following range of total estimated costs of additional outpatient healthcare visits related to CHIKV disease up to one year after illness onset: $620,400 for zero underreporting to $781,100 for 47% underreporting (S3 Table). As a result, the total estimated direct cost associated with the CHIKV outbreak in the USVI ranges from $3,536,000-$3,696,700.

**Table 4:** Direct cost estimate (2014 USD) of the chikungunya outbreak in the U.S. Virgin Islands up to 12 months after illness onset.

Outpatient			
Island	St. Croix	St. Thomas	St. John
Mean cost of a healthcare visit* ($)	600	300	234
Number of reported laboratory-positive cases + 70% of suspected not-tested cases	508	804	34
Mean number of additional healthcare visits at 1–2 months	0.5	0.5	0.5
Total cost of healthcare visits at 1–2 months ($)	152,400	120,600	3,978
Mean number of additional healthcare visits at 12 months	0.62	0.62	0.62
Total cost of healthcare visits 3–12 months ($)	188,976	149,544	4,933
Cost of outpatient visits related to CHIKV up to 12 months ($)	620,400		
**Cost of acute (1–2 months) outpatient and inpatient visits related to CHIKV ($)**	**2,915,600**		
**Total direct cost estimate of the CHIKV outbreak up to 12 months ($)**	**3,536,000**		

*The mean cost of an outpatient visit associated with a suspected CHIKV cases is higher than the mean cost of a standard outpatient visit due to additional serological testing for both chikungunya and dengue fever virus.

Note: Total cost estimates were rounded to the nearest hundred.

### Total cost estimate of the 2014–2015 CHIKV outbreak

The total direct and indirect estimated cost associated with the 2014–2015 CHIKV outbreak in the USVI ranges from $14,827,500–$33,424,600 depending on the proportion of the population infected with symptomatic CHIKV, the degree of underreporting of healthcare utilization, and the proportion of cases who were employed at the time of the outbreak.

#### Years lived with disability

In addition to the indirect cost calculation, the estimated number of YLDs associated with long-term sequelae from the 2014–2015 CHIKV outbreak in the USVI was 599–1,322 when using the disability weight for post-acute effects of infectious diseases and ranged from 427–1,407 when using disability weights consistent with prior studies (Table 5).

**Table 5:** Years lived with disability due to persistent arthralgia following the chikungunya outbreak, (total U.S. Virgin Islands population = 103,574).

	Osteo-arthritis	Post-acute effects	Rheumatoid arthritis
Disability weight	0.156	0.219	0.233
Proportion of USVI population with symptomatic infection = 0.22 [28]	22,786		
Prevalence of persistent arthralgia attributable to CHIKV 6 months after illness onset* [27]	0.32 (95% CI: 0.23–0.41)		
Prevalence of persistent arthralgia attributable to CHIKV 12 months after illness onset* [27]	0.21 (95% CI: 0.11–0.31)		
^a^**Years lived with Disability**	**942**	**1,322**	**1,407**
Prevalence of persistent arthralgia attributable to CHIKV 12 months after illness onset [28]	0.12 (95% CI: 0.07–0.17)		
^b^**Years lived with Disability**	**427**	**599**	**637**

*Unadjusted for sex, age, history of arthritis.

^a^Using a persistent arthralgia estimate of 32% at 6 months and 21% at 12 months.

^b^Using a persistent arthralgia estimate of 12%.

Note: Total YLDs were rounded to the nearest whole number.

## Discussion

This study estimated the total direct and indirect cost and burden of disease associated with the 2014–2015 CHIKV outbreak in the USVI. The total estimated cost associated with the outbreak ranged from $14.8–$33.4 million, of which 12–24% was direct costs and 76–88% was indirect costs. Up to 1% of gross domestic product (GDP) in the USVI was estimated to be lost due to the CHIKV outbreak (GDP in 2014 = $3.67 billion USD [51]).

Our direct cost estimate of the outbreak in the USVI was comparable to the cost estimate of the 2005–2006 outbreak in La Réunion, ($3.5 million for 22,786 cases in the USVI [$155 per case] compared to $50.4 million for 266,000 cases in La Réunion [$189 per case]) [23,52]. Our indirect cost estimates, were also comparable when including only the proportion of the population who was employed [23]. The seroprevalence estimate of symptomatic CHIKV cases suggests that between 16–22% of the USVI population had symptomatic infection [26]. The surveillance data may not have captured many of these cases because during the height of the outbreak, hospitals and healthcare clinics reached capacity and had to turn residents away who were seeking care. Additionally, due to public health announcements in the media during the outbreak, many residents were aware of symptoms associated with infection and knew treatment for CHIKV did not exist, so they may have opted to stay home instead of seeking healthcare.

We estimated that the number of years lived with disability associated with chronic symptoms of CHIKV ranges from 427–1,407. Our YLD estimates are more conservative than the disability-adjusted life year estimates from Latin America, due to the fact that we provided a lower estimate of persistent arthralgia attributable to CHIKV illness at 12 months (21% and 12% compared to ~50% in Latin America) [24,53]. This difference is present because both studies in the USVI [27,28] subtracted the prevalence of persistent arthralgia among non-diseased individuals from the prevalence of persistent arthralgia among CHIKV cases 12 months after acute illness, whereas the study in Latin America did not [53]. The Second United States Panel on Cost-Effectiveness in Health and Medicine recommends counting both productivity costs and YLDs for an analysis from the societal perspective, based on evidence that disability weights reflect health rather than productivity [[54]]. Although their recommendation does not necessarily apply to cost-of-illness studies, two of five published CHIKV cost-of-illness studies presented both indirect costs and YLDs, while the other three studies only presented YLDs [22–24,26,47].

Certain limitations should be considered when interpreting the results of this study. The total direct and indirect estimated costs of the 2014–2015 CHIKV outbreak in the USVI may lack precision. Ambulatory service charges, absenteeism of caretakers for those who were ill due to CHIKV and additional hospitalization costs after the acute phase of illness could not be measured and were therefore not included in analysis. The analysis also does not account for the cost of individuals with symptomatic CHIKV who did not seek acute care but did seek follow-up care. The mean cost of outpatient and inpatient visits was based solely on data from JFLHMC, and does not account for varying costs from SMRC, MKCHC and private healthcare clinics. We addressed this issue by conducting a sensitivity analysis of direct costs based on the standard cost of healthcare visits at SMRC and MKCHC. Although another sensitivity analysis was conducted to account for underreporting of healthcare utilization, the true magnitude of underreporting up to 12 months after illness onset remains unknown. Additionally, there are three potential sources of bias in the estimates of disability: 1) if cases with persistent arthralgia were more likely to participate in the follow-up study, disability would be over-estimated, 2) if the cause of death among the three cases who died was primarily CHIKV, disability would be underestimated by excluding their years of life lost, and 3) there are other documented long-term sequelae associated with CHIKV disease that we did not account for, such as mental health diagnoses, that would result in an underestimation of disability [55,56]. As a result, our YLD estimates are either consistent or more conservative than previous CHIKV studies [24,26,48,56]. Lastly, although using means, instead of medians to estimate costs is standard practice in economic analysis, the estimates presented might be elevated by certain individuals who incurred higher costs than others.

Despite these limitations, this is one of the initial cost-of-illness studies that quantifies the number of years lived with disability due to long-term sequelae of CHIKV illness in the Caribbean. The results from this study highlight the substantial economic and long-term health burden of a CHIKV outbreak and provide evidence to inform policy decisions about prevention and control measures for inevitable future CHIKV outbreaks.

## Supporting information

S1 Table Eligibility and enrollment numbers of laboratory-positive cases at 1–2, 6 and 12 months after illness onset. (DOCX)

S2 Table Sensitivity analysis of direct cost estimate (2014 USD) of the acute phase of the CHIKV outbreak in the USVI where acute phase costs on St. Croix are adjusted by the relative costs of an average outpatient visits on St. Thomas and St. John. (DOCX)

S3 Table Sensitivity analysis of reporting of healthcare utilization 12 months after acute onset of CHIKV illness and associated cost estimates (2014 USD). (DOCX)

S4 Table Sensitivity analysis of indirect cost estimates (2014 USD) due to absenteeism from the chikungunya outbreak in the U.S. Virgin Islands up to 12 months after disease onset. (DOCX)

S1 Surveillance questionnaire data USVI chikungunya surveillance data from Governor Juan F. Luis Hospital and Medical Center, including cost per suspected, probable, and confirmed chikungunya case by date of symptom onset, and 1 to 2-month, 6-month, and 12 month questionnaire data. (XLSX)

S1 STROBE Checklist (DOC)
