# Supplementary material for: Estimating the cost of illness and burden of disease associated with the 2014–2015 chikungunya outbreak in the U.S. Virgin Islands
Source: PLoS Negl Trop Dis. 2019 Jul 19;13(7):e0007563. doi: 10.1371/journal.pntd.0007563 (PMC6668848; doi:10.1371/journal.pntd.0007563)
Supplement: S1 Table — (DOCX) [file pntd.0007563.s001.docx]

S1 Table: Eligibility and enrollment numbers of laboratory-positive cases at 1-2, 6 and 12 months after illness onset [27].

|  | **Total interviewed** | | |
| --- | --- | --- | --- |
|  | **1-2 Months** | **6 Months** | **12 months** |
| Eligible Individuals | 562 | 457 | 165 |
| Missing phone number | 411 | 116 | 0 |
| Phone # not-in-service or incorrect | 24 | 93 | 9 |
| Did not pick up after 3 calls | 29 | 70 | 24 |
| Refused | 11 | 11 | 4 |
| Died | 1 | 2 | 0 |
| **Total interviewed** | **86** | **165** | **128** |
