# Supplementary material for: Estimating the cost of illness and burden of disease associated with the 2014–2015 chikungunya outbreak in the U.S. Virgin Islands
Source: PLoS Negl Trop Dis. 2019 Jul 19;13(7):e0007563. doi: 10.1371/journal.pntd.0007563 (PMC6668848; doi:10.1371/journal.pntd.0007563)
Supplement: S2 Table — (DOCX) [file pntd.0007563.s002.docx]

S2 Table: Sensitivity analysis of direct cost estimate (2014 USD) of the acute phase of the CHIKV outbreak in the USVI where acute phase costs on St. Croix are adjusted by the relative costs of an average outpatient visits on St. Thomas and St. John.

|  | Outpatient | | | Inpatient | | |
| --- | --- | --- | --- | --- | --- | --- |
|  | St. Croix | St. Thomas | St. John | St. Croix | St. Thomas | St. John* |
| Mean cost of an outpatient healthcare visit ($) | 1,526.21 | 763.11 | 595.22 | 16,982.73 | 8,491.37 | - |
| Total number of outpatient reported suspected cases * 70% of suspected not-tested cases | 498 | 757 | 40 | 34 | 21 | 0 |
| Total cost of outpatient visits related to CHIKV ($) | 760,053 | 577,674 | 23,809 | 577,413 | 178,319 | 0 |
| Total cost of outpatient & inpatient visits related to CHIKV ($) | **2,117,300** | | | | | |

* Myra Keating Community Health Center (MKCHC) does not have inpatient facilities. All individuals needing inpatient services were transported to Schneider Regional Medical Center (SMRC) on St. Thomas.

Note: Total cost estimate was rounded to the nearest hundred.
