# Supplementary material for: Estimating the cost of illness and burden of disease associated with the 2014–2015 chikungunya outbreak in the U.S. Virgin Islands
Source: PLoS Negl Trop Dis. 2019 Jul 19;13(7):e0007563. doi: 10.1371/journal.pntd.0007563 (PMC6668848; doi:10.1371/journal.pntd.0007563)
Supplement: S3 Table — (DOCX) [file pntd.0007563.s003.docx]

S3 Table: Sensitivity analysis of reporting of healthcare utilization 12 months after acute onset of CHIKV illness and associated cost estimates (2014 USD).

|  | **Outpatient** | | | | | | |
| --- | --- | --- | --- | --- | --- | --- | --- |
| **Island** | | **St. Croix** | **Total Cost** | **St. Thomas** | **Total Cost** | **St. John** | **Total Cost** |
| Mean cost of a healthcare visit ($) | | 600 |  | 300 |  | 234 |  |
| Mean number of additional healthcare visits at 12 months | | 0.62 | 188,976 | 0.62 | 149,544 | 0.62 | 4,933 |
| 5% underreporting | | 0.65 | 198,120 | 0.65 | 156,780 | 0.65 | 5,171 |
| 15% underreporting | | 0.70 | 213,360 | 0.70 | 168,840 | 0.70 | 5,569 |
| 25% underreporting | | 0.78 | 237,744 | 0.78 | 188,136 | 0.78 | 6,206 |
| 35% underreporting | | 0.84 | 256,032 | 0.84 | 202,608 | 0.84 | 6,683 |
| 45% underreporting | | 0.90 | 274,320 | 0.90 | 217,080 | 0.90 | 7,160 |
| 47% underreporting | | 0.91 | 277,368 | 0.91 | 219,492 | 0.91 | 7,240 |
| **Range of cost of outpatient visits at 12 months ($)** | | **343,500 – 504,100** | | | | | |

Note: Total cost estimates were rounded to the nearest hundred.
