# Supplementary material for: Estimating the cost of illness and burden of disease associated with the 2014–2015 chikungunya outbreak in the U.S. Virgin Islands
Source: PLoS Negl Trop Dis. 2019 Jul 19;13(7):e0007563. doi: 10.1371/journal.pntd.0007563 (PMC6668848; doi:10.1371/journal.pntd.0007563)
Supplement: S4 Table — (DOCX) [file pntd.0007563.s004.docx]

S4 Table: Sensitivity analysis of indirect cost estimates (2014 USD) due to absenteeism from the chikungunya outbreak in the U.S. Virgin Islands up to 12 months after disease onset.

| Time period after acute illness | 1-2 Months | | | 3-6 Months | | | 7-12 Months | | |
| --- | --- | --- | --- | --- | --- | --- | --- | --- | --- |
| Median number of work days missed | 4.5 | | | 0.5 | | | 0 | | |
| Mean number of work days missed | 5.6 | | | 2.2 | | | 1.2 | | |
| Mean number of work hours missed | 44.6 | | | 17 | | | 9.3 | | |
| Island | St. Thomas | St. Croix | St. John | St. Thomas | St. Croix | St. John | St. Thomas | St. Croix | St. John |
| Average Hourly Wage ($) [28] | 18.51 | 18.43 | 16.00 | 18.51 | 18.43 | 16.00 | 18.51 | 18.43 | 16.00 |
| Number of estimated cases by island when proportion of population with symptomatic infection=0.16 | 8,041 | 7,884 | 647 | 8,041 | 7,884 | 647 | 8,041 | 7,884 | 647 |
| Total value of time lost by island when proportion of population with symptomatic infection=0.16 ($) | 6,632,262 | 6,474,662 | 461,285 | 2,560,029 | 2,499,196 | 178,054 | 1,381,225 | 1,348,404 | 96,067 |
| Indirect cost attributable to the CHIKV outbreak for USVI population ($) | **21,631,200** | | | | | | | | |
| Total wages lost by island among all employed when proportion of population with symptomatic infection=0.16 ($) | 3,462,041 | 3,379,774 | 240,791 | 1,336,335 | 1,304,581 | 92,944 | 720,999 | 703,867 | 50,147 |
| Indirect cost attributable to the CHIKV outbreak for USVI population reported to be employed* ($) | **11,291,500** | | | | | | | | |

*52.2% of the U.S. Virgin Islands population was employed as of 2010 [38].

Note: Total cost estimates were rounded to the nearest hundred.
